# Modelling the impact of climatic and environmental variables on malaria incidence in Tanzania: Implications for achieving the WHO’s 2030 Targets

**DOI:** 10.1371/journal.pgph.0005075

**Published:** 2025-08-20

**Authors:** Angelina Mageni Lutambi, Basiliana Emidi, Fredrick George Mbuya, Michael Ryoba, Thadei Damas Sagamiko, Alfred Kisuda Hugo, Isambi Sailon Mbalawata

**Affiliations:** 1 Dodoma Medical Research Centre, National Institute for Medical Research, Dodoma, Tanzania; 2 Department of Mathematics, Physics and Informatics, Dar es Salaam University College of Education, University of Da es Salaam, Dar es Salaam, Tanzania; 3 Department of Mathematics and Statistics, University of Dodoma, Dodoma, Tanzania; 4 African Institute for Mathematical Sciences Research and Innovation Centre, Kigali, Rwanda; University of Embu, KENYA

## Abstract

Malaria remains a significant public health challenge, particularly among vulnerable populations in high-burden countries like Tanzania. Despite progress in reducing malaria incidence, climatic and environmental condition variability has led to uneven reductions, hindering the achievement of the WHO 2030 targets. We assessed the impact of climatic and environmental variables on malaria incidence to better understand spatial and temporal trends and their implications for the WHO targets. We utilized geo-covariate data from the Demographic and Health surveys (DHS) program, applying a Moran’s I test for spatial autocorrelation, a geostatistical Bayesian-based model to predict malaria incidence at an unsampled locations, and calculated the percentage change in predicted incidence over a ten-year interval. The results showed that malaria incidence decreased with greater variance across Tanzania. Mean malaria incidence decreased from 0.347 (95% CI: 0.336, 0.357) in 2000 to 0.118 (95% CI: 0.114, 0.122) in 2020, relative to the increasing insecticide-treated bednets (ITNs) coverage (0.037; 95% CI: 0.036, 0.039 in 2000 to 0.496; 95% CI: 0.476, 0.517 in 2020). Malaria incidence was higher in the Lake, western, eastern and southern zones compared to others, with spatial clustering observed (Moran’s I of 0.93 in 2000, 0.87 in 2010, and 0.74 in 2020). Higher temperatures increased malaria incidence (Odds ratio (OR): 1.06; 95% credible interval (CI):1.04,1.08 and 1.13;95% CI:1.10,1.16) in 2000 and 2010, respectively). Enhanced vegetation index increased the likelihood of malaria incidence (ORs ranging from 5.28; 95% CI: 4.96,5.61) in 2000 to 6.22; 95% CI: 5.91,6.55) in 2020 and higher aridity was associated with higher malaria incidence (ORs: 1.11; 95% CI: 1.10,1.13) in 2010 and 1.07; 95% CI: 1.06,1.07) in 2020). To achieve the WHO 2030 malaria reduction targets, fine-scale and region-specific interventions are essential to mitigate the impact of climate and environmental factors on malaria incidence.

## Introduction

According to the World Malaria Report 2024, Africa accounted for two-thirds of all malaria cases and deaths worldwide [1]. Moreover, Malaria has remained a significant public health challenge, particularly in vulnerable populations living in high-burden-to-high-impact (HBHI) countries. In 2022, HBHI countries accounted for 67% of global malaria cases, with an increase from 157 million cases in 2019–167 million cases in 2022 [2], while in 2023, the global malaria burden was mostly contributed by 10 HBHI countries within the WHO African Region, accounting for 69% of estimated malaria cases and 70% of deaths [1]. Efforts to reduce malaria have intensified, guided by strategic principles and global targets, with the World Health Organization (WHO) setting ambitious 2030 targets to reduce malaria incidence and mortality by 90% [3]. Yet, malaria remains a challenge in many countries.

Tanzania, being one of the HBHI countries [4], has made significant efforts in malaria control, including the implementation of malaria burden stratification. This stratification aimed to guide control programs by developing targeted intervention packages for different strata [5]. However, the country continues to face challenges due to regional variations in malaria risk, driven primarily by differences in climatic conditions. Studies have demonstrated the critical role of climatic and environmental factors in malaria transmission [6–12], influencing the distribution, abundance, behavior of malaria vectors, and the development of malaria parasites within these mosquito vectors [13–21]. Differences in ecological settings such as highlands, coastal and semi-arid regions, have shown variations in temperature and precipitation which affects malaria incidence patterns [22]. Temperature plays a critical role in mosquito survival, biting rate and the extrinsic incubation period of the malaria parasites, with warmer temperatures generally accelerating parasites development within the mosquito [23], while rising temperatures potentially reducing longevity, overall body size, duration of the gonotrophic cycle, and reproductive capacity of *Anopheles* mosquitoes [23,24]. Additionally, elevated rearing temperatures have been reported to decrease *Anopheles gambiae* sensu lato mosquitoes susceptibility to pyrethroids and increased the expression of metabolic enzymes [25], and that future warmer climate could increase resistance of mosquitoes to insecticides and complicate malaria vector control measures [23,25,26]. Rainfall usually influences mosquito vector breeding sites availability. However, excessive rainfall sometimes flush out mosquito larvae and moderate rainfall creates suitable aquatic habitats for larval development [27–29]. Furthermore, higher Enhanced Vegetation Index (EVI) values indicate dense vegetation, which create stable microclimatic conditions that support prolonged larval development and enhance adult mosquito survival [30].

Therefore, achieving global malaria elimination targets requires understanding of theses climatic and environmental factors influencing malaria trends and malaria transmission [4]. The main objective of this study was to assess the impact of climatic and environmental variables on malaria incidence in Tanzania, with the aim of understanding spatial and temporal trends, and to inform the development of fine-scale regional-specific, climate and environmental adaptive malaria control strategies to support the achievement of the WHO 2030 malaria reduction targets.

## Materials and methods

### Study setting

Tanzania is divided into regions which are further classified into nine geographical zones. These zones group together areas with similar characteristics including weather conditions. The country is tropical and situated on the eastern coast of the Indian ocean with weather and climatic conditions favourable to malaria vectors, making malaria a public health problem. Insecticide treated bed nets is the mostly and widely used intervention against malaria with several other malaria control programs in place to mitigate the burden [31].

### Data source and study design

We used secondary geo-covariate data from the spatial data repository of the Demographic and Health surveys (DHS) program for spatial analysis [32]. Geo-covariates are external spatial covariates data that are linked to DHS survey cluster locations for use in further studies [33]. These geo-covariate datasets consist of malaria related variables, climatic and environmental data at each sampled cluster. The DHS survey is a cross-sectional survey which uses a two-stage stratified sampling method. In the first stage, the country is divided into regions, and within each region, the primary sampling units (PSUs) are selected. These PSUs form clusters of households. These cluster locations are sampled so that whatever data is extracted relative to these clusters, the data becomes nationally representative. For this particular study, we used the geo-covariate dataset extracted based on the Tanzania Demographic and Health Survey and Malaria Indicator Survey (TDHS-MIS) 2022 sampling strategy conducted between February and July 2022 [34]. The TDHS-MIS 2022 sampled 629 clusters and a total of 628 clusters were georeferenced at their centroid coordinates. Georeferencing accuracy was less than 15 meters and these clusters were randomly displaced for confidentiality, with urban clusters displacements of up to 2 kilometers and up to 10 kilometers for rural clusters.

### Outcome and covariates

The geo-covariate dataset included malaria incidence, climatic, environmental, malaria specific interventions, and other covariates at each cluster sampled during the TDHS-MIS 2022 survey. Here, malaria incidence is the average number of clinical cases of *Plasmodium falciparum* malaria per person per year at the DHS survey cluster location. These incidence and covariates data for each cluster were extracted from raster and vector data from various sources, reprojected to the same standard-based World Geodetic System 1984 coordinate system, and resampled to a uniform 5 by 5 km spatial resolution [33]. Only geospatial covariates presented in Table 1 for 2000, 2010, and 2020 were extracted for use in this study and are widely recommended in literature for their effect on malaria [6–16,18].

**Table 1:** Covariates and their descriptions, and their sources described in detail in [33].

Covariate	Description	Units/Categories	Selection by VIF^1^ (round 1)	Selection by Corr. (round 2)
Elevation	Digital Elevation Model (DEM) representing the topographic surface of the bare Earth	Meters	No	–
Aridity	Aridity index ranging from most arid to most wet	Ratio	Yes	Yes
Enhanced vegetation Index	Enhanced vegetation index value ranging from least vegetation to most vegetation	Ratio	Yes	Yes
Land surface temperature	Annual land surface temperature	Degrees Celsius	Yes	No
Maximum temperature	Maximum temperature	Degrees Celsius	Yes	Yes
Mean temperature	Mean temperature	Degrees Celsius	No	–
Minimum temperature	Minimum temperature	Degrees Celsius	No	–
Precipitation	Average precipitation (per month)	Millimeters	No	–
Rainfall	Annual rainfall	Millimeters	Yes	Yes
ITN coverage	Proportion of households owning Insecticide-treated bednets (ITNs)	Proportion	Yes	Yes

^1^VIF - Variance inflation factor.

### Covariate selection

Collinearity undermines significance of covariates and the Variance Inflation Factor (VIF) provides information on redundant variables [35]. We performed covariate selection by calculating the variance inflation factor to assess for collinearity among covariates. During this process, a stepwise selection approach was used to eliminate covariates with highest VIF until all VIFs were below 5 [35]. This elimination process resulted in a set of six covariates which included rainfall, maximum temperature, land surface temperature, enhanced vegetation index, aridity, and Insecticide-treated bednets (ITNs) coverage (Table 1). Furthermore, in order to identify the potentially highly correlated climate and environmental covariates selected based on VIF [36], these covariate-pairs were compared to the correlation between them using correlation analysis implemented and visualized in R using the *GGally* package (S1A Fig, S1B Fig and S1C Fig in S1 File). Maximum temperature and land surface temperature had a correlation of 0.61, 0.70, and 0.75 for 2000, 2010, and 2020 respectively. Land surface temperature was therefore removed from our analysis. This process was repeated for each year included in the analysis and the final set of covariates selected for further analysis included only five covariates (Table 1).

### Descriptive analysis

We conducted a descriptive analysis of malaria incidence and its associated covariates. The data, which was provided at cluster level and aggregated by year, was summarized using the mean as a primary statistic to represent the average malaria incidence and covariate values within each year. To assess temporal patterns, we calculated and plotted median values over time. Additionally, we mapped the cluster level data to visualize their spatial distribution across the country.

### Spatial autocorrelation

We used Moran’s I test to investigate the presence of spatial autocorrelation or clustering on malaria incidence, our outcome variable, a condition necessary for spatial analysis [37]. The Moran’s I test was used to assess whether the spatial distribution of malaria incidence was clustered, dispersed, or randomly distributed across the country. This index quantifies similarity in the variable of interest around neighborhoods and is given by


$$\text{I}=\frac{\text{n}\sum_{i}{\sum_{j}\omega }_{ij}\left(Y_{i}-\overset{―}{Y}\right)\left(Y_{j}-\overset{―}{Y}\right)}{\left(\sum_{i\neq j}\omega_{ij}\right)\sum_{i}{\left(Y_{i}-\overset{―}{Y}\right)}^{2}}$$ (1)

with n being the total number of clusters, $Y_{i}$ is the observed values of malaria incidence at cluster i and $\overset{―}{Y}$ is the mean of all values. $\omega_{ij}$ is the spatial weight between location i and *j*, with $\omega_{ii}=0$ and *i,j=1,2,3,…,n*. Under normality assumption and large number of clusters, the z-score statistic was calculated from $z=\frac{I-E[I]}{{Var[I]}^{1/2}}$, where E[I]=−1/(n−1) and $Var[I]=E\left[I^{2}\right]-E[I{]}^{2}$ was used to compare if any spatial pattern deviated from a random pattern. A positive Moran’s statistics close 1 indicates a strong autocorrelation of malaria incidence.

### Spatial modelling

To predict malaria incidence at unsampled locations, we applied the stochastic partial differential equation (SPDE) approach [38], implemented using the Integrated Nested Laplace Approximation (INLA) technique within the R-INLA package [39]. This method is particularly suited for modelling spatially structured data and has been widely adopted in public health research to address complex spatial dependency and make predictions [40–43]. In this modelling framework, we assumed that the number of malaria cases were given by $Y_{i}$ under the total population at risk, $N_{i}$ at cluster i for a given year. Then, conditional to the risk of malaria, the probability that ana individual experiences malaria, $P\left(s_{i}\right)$ at location, $s_{i}$, malaria cases $Y_{i}$ out of $N_{i}$were assumed to follow a binomial distribution


$$Y_{i}|{P(s}_{i})=binomial(N_{i},{P(s}_{i}))$$ (2)

with a logit link function given by


$$logit\left(P\left(s_{i}\right)\right)=\beta_{0}+\beta X\left(s_{i}\right)+\Phi (I)$$ (3)

$\beta_{0}$ is the intercept, β is a vector of coefficients of covariates $X\left(s_{i}\right)$, and $P\left(s_{i}\right)$ is the malaria risk at location, $s_{i}$. To capture spatial variability and account for spatially correlated random effects, the SPDE approach models the spatial field $\Phi (s_{i})$ as a Gaussian field that can be approximated using a Gaussian Markov random field represented by


$$\begin{array}{c} \Phi \left(s_{i}\right)\sim N(0,\sum ) \end{array}$$ (4)

where ∑ is a covariance matrix determined by a spatial correlation function. We use the Matern’s covariance function given by


$$Cov(\Phi \left(s_{i}),\Phi (s_{j}\right))=\frac{\sigma^{2}}{\text{T}(\lambda )2^{1-\lambda }}(\kappa ||{\text{s}}_{\text{i}}-s_{j}|{\left|\right)}^{\lambda }\text{K}(\kappa \left|\left|s_{i}-s_{j}\right|\right|)$$ (5)

Here, K(.) is the modified Bessel function and λ is the shape parameter defining smoothness of Φ(s), $\sigma^{2}$ is the spatial variance, κ is the range parameter which determines the distance at which the spatial correlation approaches 0.1, and ||.|| is the Euclidian distance between two locations.

Given that our data was provided in terms of incidence rates ($I_{i}$) at each cluster location for each year included in the analysis, we calculated malaria incidence cases ($Y_{i}$) from $Y_{i}=I_{i}{\times N}_{i}$ with $N_{i}$ being the total population at risk derived from the UN population count variable from the data [33]. This method was applied to predict malaria incidence at unsampled locations, both with and without incorporating covariates to assess and compare the impact of covariates on predicted malaria incidence results. To identify the best performing models, we evaluated model fit to data using the Watanabe-Akaike Information Criterion (WAIC), a widely used metric for Bayesian model comparison. WAIC balances model complexity with goodness of fit, providing an estimate of predictive accuracy of each model and a small WAIC value indicate best model prediction.

### Percentage change in predicted incidence

To monitor progress towards reaching the WHO 2030 malaria targets, we utilized the continuous surface (raster) maps of predicted malaria incidence generate through the modelling methods described above for the years 2000, 2010, and 2020 to calculate percentage change over ten-year intervals. Specifically, the percentage change in predicted malaria incidence was computed using the following formula:


$$Percentagechange=\left(\frac{{Predincidence}_{startyear}-{Predincidence}_{endyear}}{Pred{incidence}_{startyear}}\right)\times 100\%$$

For the interval between 2000 and 2010, we subtracted predicted malaria incidence for 2010 (end year) from that of 2000 (start year), and for the interval between 2010 and 2020, we subtracted the predicted malaria incidence for 2020 (end year) from that of 2010 (start year). These calculations were performed using raster operations, ensuring spatial consistency and precision across all regions. The results were visualized through spatial mapping to highlight variability in malaria incidence change across different regions. This allowed us to identify areas where progress has been slow, enabling targeted interventions and strategic decision making to support malaria control and prevention efforts.

All the statistical analyses and mapping were carried out using R 4.3.3 software.

### Ethical considerations

Our study used publicly available secondary data from the DHS program and does not require ethical clearance.

## Results

### Descriptive statistics

**Fig 1:**
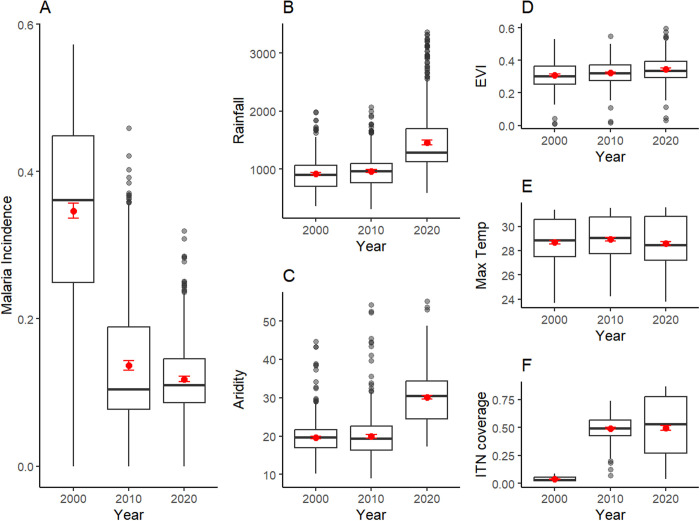
Trends in malaria incidence, climatic and environmental factors, and ITN coverage in Tanzania. Mean values and their confidence intervals are indicated as dots with error bars.

The study included 628 clusters sampled and geo-referenced during the 2022 TDHS-MIS survey. From 2000 to 2020 (Fig 1), mean malaria incidence decreased significantly from 0.347 (95% CI: 0.336, 0.357) in 2000 to 0.118 (95% CI: 0.114, 0.122) in 2020 and ITN coverage raised from 0.037 (95% CI: 0.036, 0.039) in 2000 to 0.496 (95% CI: 0.476, 0.517) in 2020. Mean maximum temperature and EVI remained relatively stable in all the years, while aridity and rainfall increased from 19.6 (95% CI: 19.3, 20.0) in 2000 to 30.1 (95% CI: 29.6, 30.5) in 2020 and 916.1 (95% CI: 896.0, 936.3) mm in 2000 to 1459.8 (95% CI: 1418.2,1501.4) mm in 2020 respectively. Similarly, the median values of malaria incidence decreased significantly over time (Fig 1). During the same period, median rainfall and aridity also increased, with maximum temperature and EVI remaining relatively unchanged. There was a noticeable increasing trend in median ITN coverage over time, starting from a low level in 2000 and increasing significantly by 2020.

**Fig 2:**
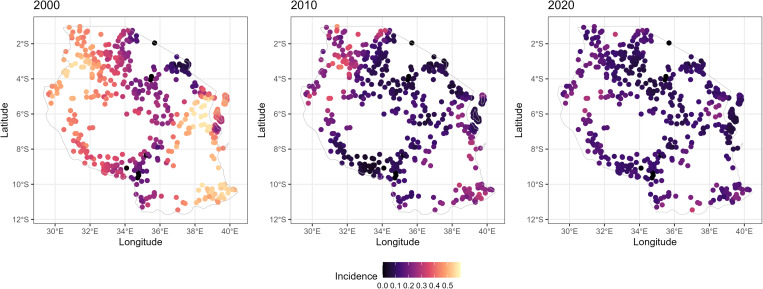
Spatial distribution of malaria incidence. Each circle represents a sampled cluster location. Column 1, 2 and 3 represent years 2000, 2010, and 2020 respectively. These maps were generated in R using a basemap shapefile obtained Natural Earth Data (https://www.naturalearthdata.com/) via the *rnaturalearth* package under a public domain license (https://www.naturalearthdata.com/about/terms-of-use/).

Malaria incidence varied across space (Fig 2), with the northwestern part of the country, around Lake Victoria recording high malaria incidence over the years. The coastal regions, particularly around Dar es Salaam, also exhibited relatively high malaria incidence. The spatial distribution of malaria incidence further improved over the years, with a significant number of regions showing very low incidence. The highest incidence areas were significantly reduced and were primarily confined to specific areas in the northwest and along the coastal regions. The distribution of maximum temperature, aridity, and EVI related to that of malaria incidence for all the years (S2A Fig and S2B Fig in S2 File) with the low malaria belt showing low maximum temperature, low values of aridity and EVI. There was small variability in rainfall across the country in 2000 and 2010, while in 2020, the Kagera, Tanga and Dar es Salaam regions had high amount of rainfall compared to other regions. ITN coverage was low in 2000 across the country, increased substantially in the high malaria areas compared to the low malaria belt in 2010. In 2020, the coverage of ITNs was lower in the central regions extending to the western and north western part compared to other parts of the country (S2C Fig in S2 File).

### Spatial clustering of malaria risk

**Fig 3:**
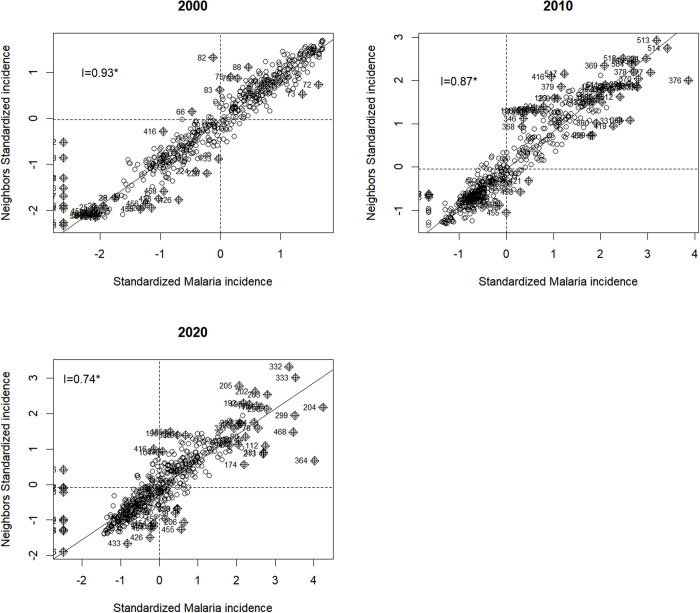
Moran’s I scatterplot of the relationship between malaria incidence in cluster and average values of neighbourhood clusters.

The Moran’s I scatterplots (Fig 3) illustrate the spatial autocorrelation of malaria incidence across the three different time periods. Each of these plots show a clear positive linear relationship between the standardized malaria incidence in an area and the average incidence in its neighboring areas with Moran’s I of 0.93 in 2000, 0.87 in 2010, and 0.74 in 2020. Areas with high malaria incidence were surrounded by other high-incidence areas, while areas with low malaria incidence were also surrounded by similarly low-incidence areas. This clustering pattern was reflected in the points distributed in the upper right (high-high) and lower left (low-low) quadrants of each plot, which indicate clusters of high and low incidence, respectively.

### Predictors of malaria incidence

We compared which model performed better than others in fitting to data. The best model for each year was then used in generating predictions in the particular year (Table 2). Results indicate that a model that combine rainfall, maximum temperature and EVI (WAIC = 15013.76) for 2000, or rainfall, maximum temperature, EVI and Aridity (WAIC = 13092.26) for 2010, or rainfall, maximum temperature, EVI, aridity and ITN (WAIC = 14477.53) for 2020 produced better fits compared to other models. Following these findings, these covariates were used in predicting malaria incidence at unsampled locations.

**Table 2:** Model selection based on best fit.

S/N	Model	WAIC^a^		
		2000	2010	2020
1	Rainfall	16347.50	14877.10	16082.20
2	Rainfall + Tmax	16305.30	14895.54	16087.56
3	Rainfall + Tmax + EVI	15013.76	13323.86	14646.82
4	Rainfall + Tmax + EVI + Aridity	15015.93	13092.26	14505.45
5	Rainfall + Tmax + EVI + Aridity + ITN	15352.82	13174.88	14477.53

^a^WAIC - Watanabe-Akaike Information Criterion. Small WAIC implies best model prediction.

Table 3 summarizes results from the best models selected to show the impact of the covariates on malaria incidence 2000, 2010, and 2020. Results show that while rainfall showed no significant effect on malaria incidence in any year, maximum temperature exhibited a variable relationship with malaria. In 2000 and 2010, higher temperatures were positively associated with increased malaria incidence (Odds ratio (OR): 1.06; 95% credible interval (CI):1.04,1.08 and 1.13; 95% CI:1.10,1.16), respectively), but in 2020, higher temperatures were associated with a decreased malaria risk (OR: 0.94; 95% CI: 0.92,0.96). EVI was consistently a strong positive predictor, with high values indicating an increased likelihood of malaria incidence in all years (ORs ranging from 5.28; 95% CI: 4.96,5.61 to 6.22; 95% CI: 5.91,6.55). Similarly, aridity was a significant predictor, with higher aridity linked to greater malaria incidence (ORs: 1.11; 95% CI: 1.10,1.13) in 2010 and 1.07; 95% CI: 1.06,1.07) in 2020). Furthermore, ITN coverage emerged as a strong predictor in 2020, with areas with higher ITN coverage having a significantly greater likelihood of high malaria incidence (ORs: 3.88; 95% CI: 3.22,4.68).

**Table 3:** Predictors of Malaria incidence as obtained from the best models.

Covariate	2000		2010		2020	
	Coeff (CI^1^)	Odds Ratio (CI)	Coeff (CI)	Odds Ratio (CI)	Coeff (CI)	Odds Ratio (CI)
Intercept	−3.13 (−3.71, −2.54)	0.04 (0.02,0.08)	−8.29 (−9.34, −7.25)	0.00 (0.00,0.001)	−3.59 (−4.27, −2.91)	0.03 (0.01, 0.06)
Rainfall	0.00 (0.00, 0.00)	1.00 (1.00,1.00)	0.00 (0.00, 0.00)	1.00 (1.00,1.00)	0.00 (0.00, 0.00)	1.00 (1.00,1.00)
Tmax	0.06 (0.04, 0.08)	1.06 (1.04,1.08)	0.12 (0.10, 0.15)	1.13 (1.10,1.16)	−0.06 (−0.08, −0.04)	0.94 (0.92,0.96)
EVI	1.83 (1.78,1.88)	6.22 (5.91,6.55)	1.66 (1.60, 1.72)	5.28 (4.96,5.61)	1.75 (1.69, 1.80)	5.74 (5.43,6.08)
Aridity	–	–	0.11 (0.10, 0.12)	1.11 (1.10,1.13)	0.06 (0.06, 0.07)	1.07 (1.06,1.07)
ITN	–	–	–	–	1.36 (1.17, 1.54)	3.88 (3.22,4.68)

^1^CI 95% Credible interval.

**Fig 4:**
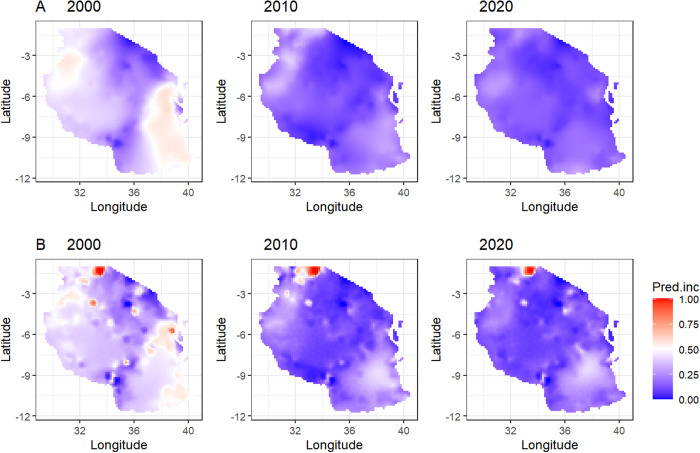
Comparison of predicted malaria incidence between model without covariates (Row A) and model with covariates-best model (Row B) and by years. These maps were generated in R using a basemap shapefile obtained Natural Earth Data (https://www.naturalearthdata.com/) via the *rnaturalearth* package.

Fig 4 illustrates the spatial distribution of predicted malaria incidence across Tanzania for the years 2000, 2010, and 2020 without covariates (Row A), and with covariates of the best models (Row B). In the year 2000, predicted malaria incidence was high in the western and the eastern regions and that highest incidence areas were more localized or clustered together. Malaria decreased in 2010, with most regions exhibiting moderate to low malaria incidence. The decreasing trend continued in 2020 with many regions exhibiting very low to moderate malaria incidence rates. Although malaria decreased significantly, still, high incidence was primarily confined to specific areas in the northwest and along the coastal and southern regions. Results also show that the inclusion of covariates could explain the differences in the distribution of malaria risk. The model without covariates (Row A) produces broad, smooth and generalized predictions with limited spatial details, while the model with covariates (Row B) captured fine-scale spatial variations, showing greater spatial heterogeneity, reflecting the impact of covariates. High risk areas aligned with areas having favorable climatic conditions for malaria, such as high maximum temperature, EVI, aridity, and rainfall (S2 Fig in S2 File). Over time, the high-risk areas appeared to contrast, reflecting the impact of interventions and varying climatic and environmental factors.

### Progress in achieving global targets

**Fig 5:**
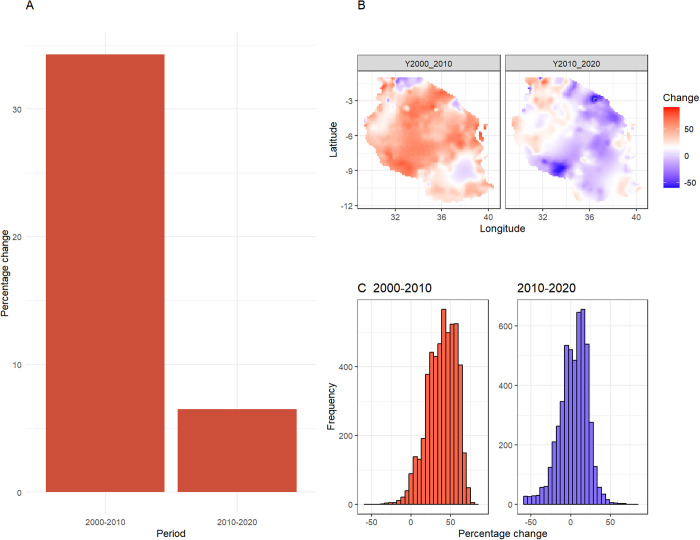
Percentage change in predicted malaria incidence. Positive % change indicates a decrease in predicted malaria incidence and negative % change indicates an increase in predicted malaria incidence. (A) Overall country mean percentage change in predicted malaria incidence; (B) Spatial distribution of percentage change in predicted malaria incidence across the country, and (C) Histograms show the distribution of the percentage change. The maps presented here were generated in R using a basemap shapefile obtained Natural Earth Data (https://www.naturalearthdata.com/) via the *rnaturalearth* package.

Fig 5 presents the percentage change in predicted malaria incidence for the two decades, 2000–2010 and 2010–2020. The reduction in predicted malaria incidence was much greater during the first decade (2000–2010) compared to the second decade (2010–2020) with percentage change of about 35% and 7% respectively (Fig 5A). Changes in predicted malaria incidence was heterogeneous across the country during the periods studied (Fig 5B). There was a widespread decrease in incidence between 2000 and 2010 with most areas showing positive percentage change. During the period between 2010 and 2020, the spatial distribution shifted, with large part of the country maintaining or had increased malaria incidence. This variability in percentage change was clearly shown by the histograms (Fig 5C) which presents the distribution of percentage change across the country. In 2000–2010, the distribution was skewed towards positive change while during 2010–2020 period, the distribution was nearly normally distributed.

## Discussion

This study assessed the impact of climatic and environmental variables on malaria to understand the spatial and temporal trends to inform development of regional-specific, climatic adaptive interventions to support achieving the WHO malaria reduction targets. Between 2000 and 2020, malaria incidence in Tanzania varied spatially, influenced by climatic and environmental factors, and interventions. Enhanced vegetation index, maximum temperature and aridity were significant predictors of malaria incidence, while rainfall, however, had no significant effect on malaria incidence in any year. In addition, ITNs coverage as part of the malaria vector control interventions was significantly associated with presence of malaria. A consistent spatial clustering of malaria incidence was observed where areas with similar values were clustered together, with low incidence areas being along the low temperature belt which originate from the northern zone to southern highlands through the central part of the country. Furthermore, inclusion of climatic variables in predictions captured fine-scale spatial variations, showing greater spatial heterogeneity and high-risk areas aligned with regions having favorable climatic conditions for malaria. Tracking change in malaria indicated that predicted malaria incidence decreased significantly during the 2000–2010 period but slowed down during the 2010–2020 period. This decrease was heterogeneous across the country during the period studied.

The finding that EVI was a significant predictor of malaria across all years aligns with other studies conducted in tropical regions [44–47]. EVI has been linked to malaria risk due to its influence on mosquito habitats. Studies in Vietnam and Gabon reported that higher EVI values were significantly associated with increased malaria risk, as dense vegetation offers favourable conditions for mosquito breeding [48,49]. Areas with higher vegetation cover support larval development and enhance the survival of adult mosquitoes, thereby contributing to increased malaria transmission [30,49]. Similarly, a study in Zambia found that environmental metrics such as EVI, topographic position index, and topographical wetness index remained constant over time and were associated with a higher likelihood of detecting malaria infections during case investigations [50]. The significancy of aridity in predicting malaria incidence is supported by evidence from Nigeria and Ghana, where increased aridity was associated with a higher risk of malaria infection [41,51]. Another study in Gabon included aridity as a significant variable in their final model, indicating its relevance in malaria transmission dynamics [48].

Similar to other studies [8,13,52], maximum temperature was positively associated with malaria risk in 2000 and 2010, as higher temperatures within suitable ranges can accelerate parasite development within mosquitoes [49]. However, a negative association between maximum temperature and malaria observed in 2020 highlighted potential changes in malaria transmission dynamics due to temperature changes. This finding may reflect the complex relationship between temperature and malaria transmission where extremely high temperatures can exceed the optimal range for mosquito survival or parasite development [41]. Malaria incidence was lower in the low-temperature belt, corresponding to higher elevations [5]. This was consistent with other studies which have shown that both temperature and malaria incidence decrease with increased elevation [53]. Our results on the impact of temperature on malaria is supported by available evidence which have shown that temperature influences malaria transmission by affecting the development rate of mosquito larvae and the survival rate of adult mosquitoes [54], and that warmer temperatures provide a favourable environment for adult female mosquitoes to feed more frequently, digest blood more rapidly, and the *plasmodium* parasite matures more quickly within the female mosquitoes [55]. Despite the fact that rainfall is positively associated with malaria in specific areas of Tanzania [56] and that rainfall play a crucial role in creating the necessary conditions for surface water accumulation, which serves as breeding sites for mosquitoes [14,51], our results that rainfall did not significantly affect malaria incidence, suggests that other factors played a dominant role in shaping malaria patterns in the country.

Similar to another study in Gabon which demonstrated that increased ITN coverage was significantly associated with a reduction in malaria prevalence [48], our descriptive and spatial temporal results also indicated that ITNs were associated with malaria reduction, with their impact becoming more evident in later years. The widespread use of insecticide-treated bednets, along with other vector control interventions, has played a crucial role in reducing malaria incidence [57,58]. The expansion of ITN coverage in Tanzania has been driven by various net distribution and delivery initiatives, including social marketing, subsidized vouchers, mass campaigns targeting children under five, universal coverage campaigns, antenatal care initiatives, the private commercial market, and school net distribution programs [59–62]. All of these initiatives increased ITNs coverage, thus, impacting malaria. Although ITNs are proven to reduce malaria transmission by killing or repelling mosquitoes, our 2020 spatial model showed that high ITN coverage was associated with high malaria incidence. Similar findings have been reported elsewhere. In Ghana, higher ITN coverage correlated with increased malaria prevalence [41], in Nigeria, ITN distribution was positively associated with malaria prevalence, suggesting that this may reflect targeted ITN distribution to high-risk areas [63]. In northwest Tanzania, high community-level ITN coverage did not reduce malaria prevalence due to confounding factors like socioeconomic status and housing conditions [64]. All of this evidence likely reflects a complex interplay of programmatic targeting, behavioral, and environmental factors. Areas with a high malaria burden are often prioritized for ITN distribution as part of targeted national malaria control strategies, thus, increased ITN coverage. This phenomenon where disease drives intervention coverage can create a positive association between ITN coverage and malaria.

Spatial analysis revealed consistent geographic variability in malaria incidence over the study period, with high-incidence regions predominantly located in the northwestern areas, around Lake Victoria, parts of the western regions, as well as coastal regions and around the Rufiji basin. Over time, these high-incidence pockets contracted significantly, becoming more localized by 2020. While this reduction can be attributed to interventions [58,65,66], spatial variability in climatic and environmental factors created heterogeneity in the distribution of mosquito resources, of which theoretical modelling studies has shown to impact the mosquito populations [67]. The Moran’s I analysis confirmed consistent spatial clustering of malaria incidence, where high-incidence areas were surrounded by other high-incidence areas, aligning with studies conducted in Kenya and Rwanda [68,69] which emphasized the importance of geographically targeted interventions to disrupt transmission clusters. Predicted malaria incidence maps highlighted the importance of including covariates in modeling malaria risk. Models incorporating climatic and environmental variables and ITN coverage provided fine-scale spatial details, capturing heterogeneity. High-risk areas were concentrated in regions with favourable climatic and/or environmental conditions for malaria, and their contraction over time aligns with the observed reductions in incidence.

In terms of regional focus, the persistent high-incidence areas in the northwest and some coastal regions highlighted the need for targeted interventions in these regions. Understanding the specific local factors contributing to sustained transmission in these areas, such as environmental conditions, vector species, and human behaviors, is crucial for developing effective strategies [70]. A significant reduction in malaria incidence was observed between 2000 and 2010, likely due to the widespread adoption of rapid diagnostic tests and interventions [57,58,71]. However, progress slowed considerably from 35% between 2000 and 2010 to only a 7% reduction in predicted incidence during 2010 and 2020 period. This slow pace in malaria reduction is attributed to variability in climatic conditions [72] and the rapid increase in insecticide resistance among malaria mosquito vectors [73,74], challenging future malaria control strategies and public health interventions. To achieve the WHO 2030 malaria targets requires data-driven, climate-sensitive interventions. This study shows that using spatial modeling by incorporating climatic and environmental factors could help in identifying fine scale high-risk areas for planning and resource allocation. As evidenced by our results, scaling up ITN coverage coupled with climate informed decisions is critical to achieving further reductions in malaria, especially in areas where climatic conditions favour the transmission potential.

Although the malaria control program has created malaria stratification in the country for targeted interventions [5], the fine-scale malaria risk heterogeneity observed in this study suggest that within the large malaria strata created requires concerted malaria control efforts to achieve the WHO 2030 malaria reduction targets. Importantly, the persistence of within-strata fine-scale heterogeneity suggest that even within the low malaria strata, there are areas that face more significant high malaria incidence. These within-strata disparities indicate that not all areas within the strata have equal risk as some may still have relatively higher risk than others, due to differences in factors such as EVI. These finding highlight the need for more targeted and finely tuned interventions that address the unique fine-scale malaria risk differences. Programs aimed at reducing malaria in the country must therefore consider the diverse needs of subpopulations within broader climatic and environmental aspects, ensuring that most areas receive adequate interventions. The identification of environmental predictors such as EVI and aridity could guide spatial targeting of malaria interventions. Regions with persistent high vegetation and aridity levels can benefit from prioritized intervention, enhanced vector surveillance or tailored community health education campaigns. Integrating model outputs into national malaria control programs could boost efficiency and effectiveness of intervention planning and resource allocation.

The study results showcase several strengths that enhance its robustness and relevance in understanding malaria dynamics at finer spatial scales and informing policy. The use of data spanning two decades (2000–2020) provides a detailed temporal analysis, enabling the identification of trends, the effectiveness of malaria interventions over time, and impact of climatic and environmental conditions. With a large spatial coverage of 628 geo-referenced clusters from the TDHS-MIS 2022, the study ensures a representative dataset that captures local variations in malaria incidence across Tanzania. On the other hand, the study utilized secondary data from the DHS geo-covariate dataset to derive geo-referenced malaria incidence and ITN coverage, alongside remote sensing and meteorological data for climatic and environmental covariates. While these sources provided valuable insights, the aggregation of data at cluster levels may not fully reflect micro-environmental or household-level malaria risk variations. Furthermore, our study did not account for the seasonal variations in the covariates, a critical aspect in malaria transmission dynamics. Seasonal fluctuations in these climatic variables can influence mosquito breeding, survival rates, and parasite development, ultimately impact malaria incidence. By averaging climatic variables, we capture their overall impact over time, but this approach may overlook short-term variations that could provide deeper insights into malaria transmission patterns. Future work incorporating seasonal trends in climate data could offer a more detailed understanding of the relationship between climate and malaria risk. Additionally, our model outputs were not externally validated using independent data sources such as entomological, clinical, or surveillance records, which represents a limitation of this study. Moreover, our analysis did not include potential confounders such as access to health services and human mobility or travel. These factors are known to influence malaria transmission in such a way that limited access to health services may lead to underreporting of malaria cases, while mobility can alter exposure risk to malaria vectors [75–79]. The absence of these variables may introduce residual confounding and should be considered when interpreting our findings. Future studies could incorporate these factors to strengthen the predictive power of the models. Furthermore, the modelling approach used assumes spatial stationarity and Gaussian priors which may not fully capture the complex and potentially non-stationary nature of malaria transmission. Also, while the binomial model was appropriate for modeling incidence as a probability based on a known population at risk, it assumes independence of infection events within clusters and does not account for possible overdispersion or local clustering.

## Conclusion

This study has provided valuable insights into the spatial and temporal influence of climatic and environmental factors on malaria incidence in Tanzania. Climatic and environmental variables strongly impacted malaria. There was significant within-country spatial heterogeneity in malaria risks with some areas demonstrating notable progress, while others experienced slow progress in malaria reduction. Following this, areas with lower incidence may still require vigilance to prevent resurgence, particularly in the context of changing climatic and environmental conditions that can influence mosquito breeding grounds and malaria parasite transmission dynamics. Further research is needed to explore the specific local factors contributing to malaria persistence in certain areas and to develop tailored interventions which could integrate climatic and environmental characteristics.

## Supporting information

S1 File S1A Fig. Pairwise correlation of covariates for 2000. S1B Fig. Pairwise correlation of covariates for 2010. S1C Fig. Pairwise correlation of covariates for 2020.
(DOCX)

S2 File S2A Fig. Spatial distribution of covariates. Each circle represents a sampled cluster location for Rainfall (Row 1), and Maximum temperature (Row 2). S2B Fig. Spatial distribution of covariates. Each circle represents a sampled cluster location for Aridity (Row 1), and EVI (Row 2). S2C Fig: Spatial distribution of ITN coverage. Each circle represents a sampled cluster location for ITN coverage.
(DOCX)

## References

[1] World Health Organization. World Malaria Report 2024: addressing inequity in the global malaria response. Geneva: World Health Organization; 2024.

[2] WHO. World malaria report 2023. 2023 [cited 2024 May 5]. Available from: https://www.who.int/publications-detail-redirect/9789240086173

[3] World Health Organization. Global technical strategy for malaria 2016-2030. Geneva: World Health Organization; 2015. pp. 29. [cited 2024 May 5]. Available from: https://iris.who.int/handle/10665/176712

[4] World Health Organization. High burden to high impact: a targeted malaria response. 2018. [cited 2024 Nov 15]. Available from: https://www.who.int/publications/i/item/WHO-CDS-GMP-2018.25

[5] Thawer SG, Chacky F, Runge M, Reaves E, Mandike R, Lazaro S, et al. Sub-national stratification of malaria risk in mainland Tanzania: a simplified assembly of survey and routine data. Malar J. 2020;19(1):177. doi: 10.1186/s12936-020-03250-4 32384923 PMC7206674

[6] Zhao X, Chen F, Feng Z, Li X, Zhou X-H. Characterizing the effect of temperature fluctuation on the incidence of malaria: an epidemiological study in south-west China using the varying coefficient distributed lag non-linear model. Malar J. 2014;13:192. doi: 10.1186/1475-2875-13-192 24886630 PMC4050477

[7] Yamana TK, Eltahir EAB. Projected impacts of climate change on environmental suitability for malaria transmission in West Africa. Environ Health Perspect. 2013;121(10):1179–86. doi: 10.1289/ehp.1206174 24043443 PMC3801455

[8] Wang Z, Liu Y, Li Y, Wang G, Lourenço J, Kraemer M. The relationship between rising temperatures and malaria incidence in Hainan, China, from 1984 to 2010: a longitudinal cohort study. Lancet Planet Health. 2022;6(4):e350–8. 10.1016/S2542-5196(22)00039-0 35397223

[9] Qiao L, Yaping W, Jie D, Wenxin Y, Chenyuan Q, Min D, Min L, et al. Association of temperature and precipitation with malaria incidence in 57 countries and territories from 2000 to 2019: A worldwide observational study. J Glob Health. 2024:14:04021. doi: 10.7189/jogh.14.04021 38385445 PMC10882640

[10] Parham PE, Pople D, Christiansen-Jucht C, Lindsay S, Hinsley W, Michael E. Modeling the role of environmental variables on the population dynamics of the malaria vector Anopheles gambiae sensu stricto. Malar J. 2012;11:271. doi: 10.1186/1475-2875-11-271 22877154 PMC3496602

[11] Nyawanda BO, Beloconi A, Khagayi S, Bigogo G, Obor D, Otieno NA, et al. The relative effect of climate variability on malaria incidence after scale-up of interventions in western Kenya: A time-series analysis of monthly incidence data from 2008 to 2019. Parasite Epidemiol Control. 2023;21:e00297. doi: 10.1016/j.parepi.2023.e00297 PMC10068258 37021322

[12] Mboera LEG, Mayala BK, Kweka EJ, Mazigo HD. Impact of climate change on human health and health systems in Tanzania: a review. Tanzan J Health Res. 2011;13(5):407–26. doi: 10.4314/thrb.v13i5.10 26591995

[13] Mohammadkhani M, Khanjani N, Bakhtiari B, Sheikhzadeh K. The relation between climatic factors and malaria incidence in Kerman, South East of Iran. Parasite Epidemiol Control. 2016;1(3):205–10. doi: 10.1016/j.parepi.2016.06.001 29988199 PMC5991842

[14] Dabaro D, Birhanu Z, Negash A, Hawaria D, Yewhalaw D. Effects of rainfall, temperature and topography on malaria incidence in elimination targeted district of Ethiopia. Malar J. 2021;20(1):104. doi: 10.1186/s12936-021-03641-1 33608004 PMC7893867

[15] Chuang T-W, Soble A, Ntshalintshali N, Mkhonta N, Seyama E, Mthethwa S, et al. Assessment of climate-driven variations in malaria incidence in Swaziland: toward malaria elimination. Malar J. 2017;16(1):232. doi: 10.1186/s12936-017-1874-0 28571572 PMC5455096

[16] Kripa PK, Thanzeen PS, Jaganathasamy N, Ravishankaran S, Anvikar AR, Eapen A. Impact of climate change on temperature variations and extrinsic incubation period of malaria parasites in Chennai, India: implications for its disease transmission potential. Parasit Vectors. 2024;17(1):134. doi: 10.1186/s13071-024-06165-0 38491547 PMC11334410

[17] Paaijmans KP, Read AF, Thomas MB. Understanding the link between malaria risk and climate. Proc Natl Acad Sci U S A. 2009;106(33):13844–9. doi: 10.1073/pnas.0903423106 19666598 PMC2720408

[18] Paaijmans KP, Blanford S, Bell AS, Blanford JI, Read AF, Thomas MB. Influence of climate on malaria transmission depends on daily temperature variation. Proc Natl Acad Sci. 2010;107(34):15135–9. doi: 10.1073/pnas.1006422107 20696913 PMC2930540

[19] Abbasi M, Foroushani AR, Jafari-Koshki T, Pakdad K, Vatandoost H, Hanafi-Bojd AA. The impact of climatic variables on the population dynamics of the main malaria vector, Anopheles stephensi Liston (Diptera: Culicidae), in southern Iran. Asian Pac J Trop Med. 2020;13(10):448.

[20] Simon-Oke IA, Olofintoye LK. The effect of climatic factors on the distribution and abundance of mosquito vectors in Ekiti State. J Biol Agric Healthc. 2015;5(9).

[21] Minakawa N, Sonye G, Mogi M, Githeko A, Yan G. The effects of climatic factors on the distribution and abundance of malaria vectors in Kenya. J Med Entomol. 2002;39(6):833–41. doi: 10.1603/0022-2585-39.6.833 12495180

[22] Drake JM, Beier JC. Ecological niche and potential distribution of Anopheles arabiensis in Africa in 2050. Malar J. 2014;13:213. doi: 10.1186/1475-2875-13-213 24888886 PMC4066281

[23] Agyekum TP, Botwe PK, Arko-Mensah J, Issah I, Acquah AA, Hogarh JN. A systematic review of the effects of temperature on Anopheles mosquito development and survival: implications for malaria control in a future warmer climate. Int J Environ Res Public Health. 2021;18(14):7255. doi: 10.3390/ijerph18147255 34299706 PMC8306597

[24] Bayoh MN, Lindsay SW. Temperature-related duration of aquatic stages of the Afrotropical malaria vector mosquito Anopheles gambiae in the laboratory. Med Vet Entomol. 2004;18(2):174–9. doi: 10.1111/j.0269-283X.2004.00495.x 15189243

[25] Agyekum TP, Arko-Mensah J, Botwe PK, Hogarh JN, Issah I, Dadzie SK, et al. Relationship between temperature and Anopheles gambiae sensu lato mosquitoes’ susceptibility to pyrethroids and expression of metabolic enzymes. Parasit Vectors. 2022;15(1):163. doi: 10.1186/s13071-022-05273-z 35527275 PMC9080126

[26] Huang J, Walker ED, Vulule J, Miller JR. Daily temperature profiles in and around Western Kenyan larval habitats of Anopheles gambiae as related to egg mortality. Malar J. 2006;5:87. doi: 10.1186/1475-2875-5-87 17038186 PMC1617108

[27] Rozendaal JA. Relations between Anopheles darlingi breeding habitats, rainfall, river level and malaria transmission rates in the rain forest of Suriname. Med Vet Entomol. 1992;6(1):16–22. doi: 10.1111/j.1365-2915.1992.tb00029.x 1600221

[28] Koenraadt CJM, Githeko AK, Takken W. The effects of rainfall and evapotranspiration on the temporal dynamics of *Anopheles gambiae* s.s. and *Anopheles arabiensis* in a Kenyan village. Acta Trop. 2004;90(2):141–53. doi: 10.1016/j.actatropica.2003.11.007 15177140

[29] Paaijmans KP, Wandago MO, Githeko AK, Takken W. Unexpected high losses of Anopheles gambiae larvae due to rainfall. PLoS One. 2007;2(11):e1146. doi: 10.1371/journal.pone.0001146 17987125 PMC2063461

[30] Longo-Pendy NM, Sevidzem SL, Makanga BK, Ndotit-Manguiengha S, Boussougou-Sambe ST, Obame Ondo Kutomy P, et al. Assessment of environmental and spatial factors influencing the establishment of Anopheles gambiae larval habitats in the malaria endemic province of Woleu-Ntem, northern Gabon. Malar J. 2024 ;23(1):158. doi: 10.1186/s12936-024-04980-5 38773512 PMC11106858

[31] National Malaria Control Program. National Malaria Strategic Plan 2021-2025: Transitioning to Malaria elimination in Phases. Ministry of Health, Community Development, Gender; 2020.

[32] Spatial Data Repository - Resources. [cited 2024 Jul 18]. Available from: https://spatialdata.dhsprogram.com/resources/

[33] Mayala B, Donohue R. The DHS Program Geospatial Covariate Datasets Manual. 3rd ed. Rockville, Maryland, USA: ICF; 2022.

[34] Mainland M of H [Tanzania, Health [Zanzibar M of, Statistics (NBS) NB of, Statistician (OCGS) O of the CG, ICF. Tanzania demographic and health survey 2022 - final report. 2023 [cited 2024 May 31]; Available from: https://dhsprogram.com/publications/publication-FR382-DHS-Final-Reports.cfm

[35] Kim JH. Multicollinearity and misleading statistical results. Korean J Anesthesiol. 2019;72(6):558–69. doi: 10.4097/kja.19087 31304696 PMC6900425

[36] Mukaka MM. Statistics corner: A guide to appropriate use of correlation coefficient in medical research. Malawi Med J. 2012;24(3):69–71. 23638278 PMC3576830

[37] Chen Y. Spatial autocorrelation equation based on Moran’s index. Sci Rep. 2023;13(1):19296. doi: 10.1038/s41598-023-45947-x 37935705 PMC10630413

[38] Krainski E, Gómez-Rubio V, Bakka H, Lenzi A, Castro-Camilo D, Simpson D. Advanced Spatial Modeling with Stochastic Partial Differential Equations Using R and INLA. New York: Chapman and Hall/CRC; 2018.

[39] Rue H, Martino S, Chopin N. Approximate Bayesian inference for latent Gaussian models by using integrated nested Laplace approximations. J R Stat Soc Ser B Stat Methodol. 2009;71(2):319–92.

[40] Kang SY, Battle KE, Gibson HS, Ratsimbasoa A, Randrianarivelojosia M, Ramboarina S, et al. Spatio-temporal mapping of Madagascar’s Malaria Indicator Survey results to assess Plasmodium falciparum endemicity trends between 2011 and 2016. BMC Med. 2018;16(1):71. doi: 10.1186/s12916-018-1060-4 29788968 PMC5964908

[41] Aheto JMK. Mapping under-five child malaria risk that accounts for environmental and climatic factors to aid malaria preventive and control efforts in Ghana: Bayesian geospatial and interactive web-based mapping methods. Malar J. 2022;21(1):384. doi: 10.1186/s12936-022-04409-x 36522667 PMC9756577

[42] Aheto JMK, Menezes LJ, Takramah W, Cui L. Modelling spatiotemporal variation in under-five malaria risk in Ghana in 2016–2021. Malar J. 2024;23(1):102. doi: 10.1186/s12936-024-04918-x 38594716 PMC11005246

[43] Weiss DJ, Lucas TCD, Nguyen M, Nandi AK, Bisanzio D, Battle KE, et al. Mapping the global prevalence, incidence, and mortality of Plasmodium falciparum, 2000–17: a spatial and temporal modelling study. Lancet. 2019;394(10195):322–31. doi: 10.1016/S0140-6736(19)31097-9 31229234 PMC6675740

[44] Okunlola O, Oloja S, Ebiwonjumi A, Oyeyemi O. Vegetation index and livestock practices as predictors of malaria transmission in Nigeria. Sci Rep. 2024;14(1):9565. doi: 10.1038/s41598-024-60385-z 38671079 PMC11053042

[45] Midekisa A, Senay G, Henebry GM, Semuniguse P, Wimberly MC. Remote sensing-based time series models for malaria early warning in the highlands of Ethiopia. Malar J. 2012;11:165. doi: 10.1186/1475-2875-11-165 22583705 PMC3493314

[46] Kigozi R, Zinszer K, Mpimbaza A, Sserwanga A, Kigozi SP, Kamya M. Assessing temporal associations between environmental factors and malaria morbidity at varying transmission settings in Uganda. Malar J. 2016;15(1):511. doi: 10.1186/s12936-016-1549-2 27756304 PMC5070351

[47] Ricotta EE, Frese SA, Choobwe C, Louis TA, Shiff CJ. Evaluating local vegetation cover as a risk factor for malaria transmission: a new analytical approach using ImageJ. Malar J. 2014;13:94. doi: 10.1186/1475-2875-13-94 24620929 PMC4007634

[48] Mougeni F, Lell B, Kandala NB, Chirwa T. Bayesian spatio-temporal analysis of malaria prevalence in children between 2 and 10 years of age in Gabon. Malar J. 2024;23(1):57. doi: 10.1186/s12936-024-04880-8 38395876 PMC10893641

[49] Tam LT, Thinkhamrop K, Suttiprapa S, Clements ACA, Wangdi K, Suwannatrai AT. Bayesian spatio-temporal modelling of environmental, climatic, and socio-economic influences on malaria in Central Vietnam. Malar J. 2024;23(1):258. doi: 10.1186/s12936-024-05074-y 39182127 PMC11344946

[50] Larsen DA, Ngwenya-Kangombe T, Cheelo S, Hamainza B, Miller J, Winters A, et al. Location, location, location: environmental factors better predict malaria-positive individuals during reactive case detection than index case demographics in Southern Province, Zambia. Malar J. 2017;16(1):18. doi: 10.1186/s12936-016-1649-z 28061853 PMC5219724

[51] Okunlola OA, Oyeyemi OT. Spatio-temporal analysis of association between incidence of malaria and environmental predictors of malaria transmission in Nigeria. Sci Rep. 2019;9(1):17500. doi: 10.1038/s41598-019-53814-x 31767899 PMC6877532

[52] Liu Q, Wang Y, Deng J, Yan W, Qin C, Du M, et al. Association of temperature and precipitation with malaria incidence in 57 countries and territories from 2000 to 2019: A worldwide observational study. J Glob Health. 2024;14:04021. doi: 10.7189/jogh.14.04021 38385445 PMC10882640

[53] Patz JA, Olson SH. Malaria risk and temperature: influences from global climate change and local land use practices. Proc Natl Acad Sci U S A. 2006;103(15):5635–6. doi: 10.1073/pnas.0601493103 16595623 PMC1458623

[54] Lindsay SW, Martens WJ. Malaria in the African highlands: past, present and future. Bull World Health Organ. 1998;76(1):33–45. 9615495 PMC2305628

[55] Githeko AK, Lindsay SW, Confalonieri UE, Patz JA. Climate change and vector-borne diseases: a regional analysis. Bull World Health Organ. 2000;78(9):1136–47. 11019462 PMC2560843

[56] Jones AE, Wort UU, Morse AP, Hastings IM, Gagnon AS. Climate prediction of El Niño malaria epidemics in north-west Tanzania. Malar J. 2007;6:162. doi: 10.1186/1475-2875-6-162 18062817 PMC2228309

[57] Kisinza WN, Nkya TE, Kabula B, Overgaard HJ, Massue DJ, Mageni Z, et al. Multiple insecticide resistance in Anopheles gambiae from Tanzania: a major concern for malaria vector control. Malar J. 2017;16(1):439. doi: 10.1186/s12936-017-2087-2 29084560 PMC5663032

[58] Kakilla C, Manjurano A, Nelwin K, Martin J, Mashauri F, Kinung’hi SM. Malaria vector species composition and entomological indices following indoor residual spraying in regions bordering Lake Victoria, Tanzania. Malar J. 2020;19(1):1–14. doi: 10.1186/s12936-020-03452-w 33115495 PMC7594290

[59] Stuck L, Chacky F, Festo C, Lutambi A, Abdul R, Greer G. Evaluation of long-lasting insecticidal net distribution through schools in Southern Tanzania. Health Policy Plan. 2022;37(2):243–54. doi: 10.1093/heapol/czab140 34918055

[60] Gingrich CD, Hanson KG, Marchant TJ, Mulligan JA, Mponda H. Household demand for insecticide-treated bednets in Tanzania and policy options for increasing uptake. Health Policy Plan. 2011;26(2):133–41. 20660208 10.1093/heapol/czq027

[61] Njau RJ, de Savigny D, Gilson L, Mwageni E, Mosha FW. Implementation of an insecticide-treated net subsidy scheme under a public-private partnership for malaria control in Tanzania – challenges in implementation. Malar J. 2009;8(1):201. doi: 10.1186/1475-2875-8-201 19698109 PMC3224907

[62] Kikumbih N, Hanson K, Mills A, Mponda H, Schellenberg JA. The economics of social marketing: the case of mosquito nets in Tanzania. Soc Sci Med. 2005;60(2):369–81. doi: 10.1016/j.socscimed.2004.05.005 15522492

[63] Okunlola OA, Oyeyemi OT, Lukman AF. Modeling the relationship between malaria prevalence and insecticide-treated bed net coverage in Nigeria using a Bayesian spatial generalized linear mixed model with a Leroux prior. Epidemiol Health. 2021;43:e2021041. doi: 10.4178/epih.e2021041 PMC8510838 34098626

[64] West PA, Protopopoff N, Rowland M, Cumming E, Rand A, Drakeley C, et al. Malaria risk factors in North West Tanzania: the effect of spraying, nets and wealth. PLoS One. 2013;8(6):e65787. doi: 10.1371/journal.pone.0065787 23762425 PMC3676352

[65] Kaufman MR, Rweyemamu D, Koenker H, Macha J. “My children and I will no longer suffer from malaria”: a qualitative study of the acceptance and rejection of indoor residual spraying to prevent malaria in Tanzania. Malar J. 2012;11:220. doi: 10.1186/1475-2875-11-220 22747610 PMC3438051

[66] Kaindoa EW, Matowo NS, Ngowo HS, Mkandawile G, Mmbando A, Finda M, et al. Interventions that effectively target Anopheles funestus mosquitoes could significantly improve control of persistent malaria transmission in south-eastern Tanzania. PLoS One. 2017;12(5):e0177807. doi: 10.1371/journal.pone.0177807 28542335 PMC5436825

[67] Lutambi AM, Penny MA, Smith T, Chitnis N. Mathematical modelling of mosquito dispersal in a heterogeneous environment. Math Biosci. 2013;241(2):198–216. doi: 10.1016/j.mbs.2012.11.013 23246807

[68] Olela SO, Makokha GL, Obiero K. Spatiotemporal Variation of Malaria Transmission in Different Altitudes of Lower Lake Victoria Basin, Kenya. Eur J Health Sci. 2022;7(6):1–24.

[69] Rubuga FK, Moraga P, Ahmed A, Siddig E, Remera E, Moirano G. Spatio-temporal dynamics of malaria in Rwanda between 2012 and 2022: a demography-specific analysis. Infect Dis Poverty. 2024;13(1):67. doi: 10.1186/s40249-024-01237-w 39278924 PMC11403800

[70] Mwalimu CD, Kiware S, Nshama R, Derua Y, Machafuko P, Gitanya P. Dynamics of malaria vector composition and Plasmodium falciparum infection in mainland Tanzania: 2017–2021 data from the national malaria vector entomological surveillance. Malar J. 2024;23(1):29. doi: 10.1186/s12936-024-04849-7 38243220 PMC10797900

[71] Kibusi SM, Kimunai E, Hines CS. Predictors for uptake of intermittent preventive treatment of malaria in pregnancy (IPTp) in Tanzania. BMC Public Health. 2015;15:540. doi: 10.1186/s12889-015-1905-0 26049737 PMC4458339

[72] Li J, Docile HJ, Fisher D, Pronyuk K, Zhao L. Current Status of Malaria Control and Elimination in Africa: Epidemiology, Diagnosis, Treatment, Progress and Challenges. J Epidemiol Glob Health. 2024;14(3):561–79. doi: 10.1007/s44197-024-00228-2 38656731 PMC11442732

[73] Nkya TE, Akhouayri I, Kisinza W, David J-P. Impact of environment on mosquito response to pyrethroid insecticides: facts, evidences and prospects. Insect Biochem Mol Biol. 2013;43(4):407–16. doi: 10.1016/j.ibmb.2012.10.006 23123179

[74] Kisinza WN, Nkya TE, Kabula B, Overgaard HJ, Massue DJ, Mageni Z, et al. Multiple insecticide resistance in Anopheles gambiae from Tanzania: a major concern for malaria vector control. Malar J. 2017;16(1):439. doi: 10.1186/s12936-017-2087-2 29084560 PMC5663032

[75] Carrasco-Escobar G, Matta-Chuquisapon J, Manrique E, Ruiz-Cabrejos J, Barboza JL, Wong D, et al. Quantifying the effect of human population mobility on malaria risk in the Peruvian Amazon. R Soc Open Sci. 2022;9(7):211611. doi: 10.1098/rsos.211611 35875474 PMC9297009

[76] Porter TR, Finn TP, Silumbe K, Chalwe V, Hamainza B, Kooma E, et al. Recent travel history and Plasmodium falciparum malaria infection in a region of heterogenous transmission in Southern Province, Zambia. Am J Trop Med Hyg. 2020;103(2_Suppl):74–81. doi: 10.4269/ajtmh.19-0660 32618250 PMC7416974

[77] Abdalal SA, Yukich J, Andrinopoulos K, Alghanmi M, Wakid MH, Zawawi A. Livelihood activities, human mobility, and risk of malaria infection in elimination settings: a case–control study. Malar J. 2023;22(1):53. doi: 10.1186/s12936-023-04470-0 36782234 PMC9926773

[78] Gomez J, Grosso A, Guzman-Guzman M, Garcia Castillo S, Castro MC, Torres K, et al. Human mobility and malaria risk in peri-urban and rural communities in the Peruvian Amazon. PLoS Negl Trop Dis. 2025;19(1):e0012058. doi: 10.1371/journal.pntd.0012058 39761298 PMC11737848

[79] Mboera LEG, Makundi EA, Kitua AY. Uncertainty in Malaria Control in Tanzania: Crossroads and Challenges for Future Interventions. In: Defining and Defeating the Intolerable Burden of Malaria III: Progress and Perspectives: Supplement to Volume 77(6) of American Journal of Tropical Medicine and Hygiene. American Society of Tropical Medicine and Hygiene; 2007 [cited 2025 Jan 31]. Available from: https://www.ncbi.nlm.nih.gov/books/NBK1714/18165482

