# Supplementary material for: Modelling the impact of climatic and environmental variables on malaria incidence in Tanzania: Implications for achieving the WHO’s 2030 Targets
Source: PLOS Glob Public Health. 2025 Aug 20;5(8):e0005075. doi: 10.1371/journal.pgph.0005075 (PMC12367119; doi:10.1371/journal.pgph.0005075)
Supplement: S1 File — S1B Fig. Pairwise correlation of covariates for 2010. S1C Fig. Pairwise correlation of covariates for 2020. (DOCX) [file pgph.0005075.s001.docx]

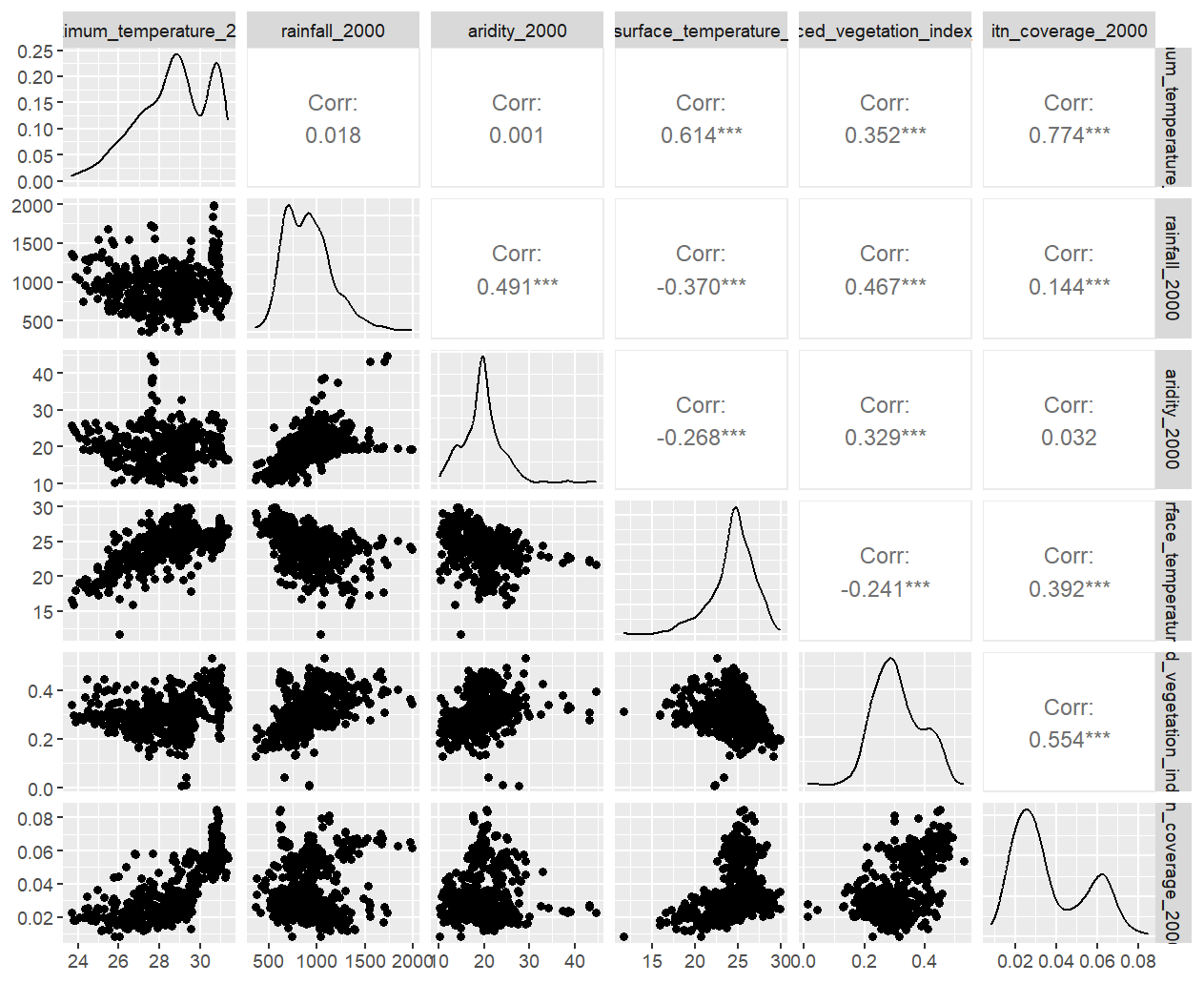


**S1A Fig.** Pairwise correlation of covariates for 2000.

**
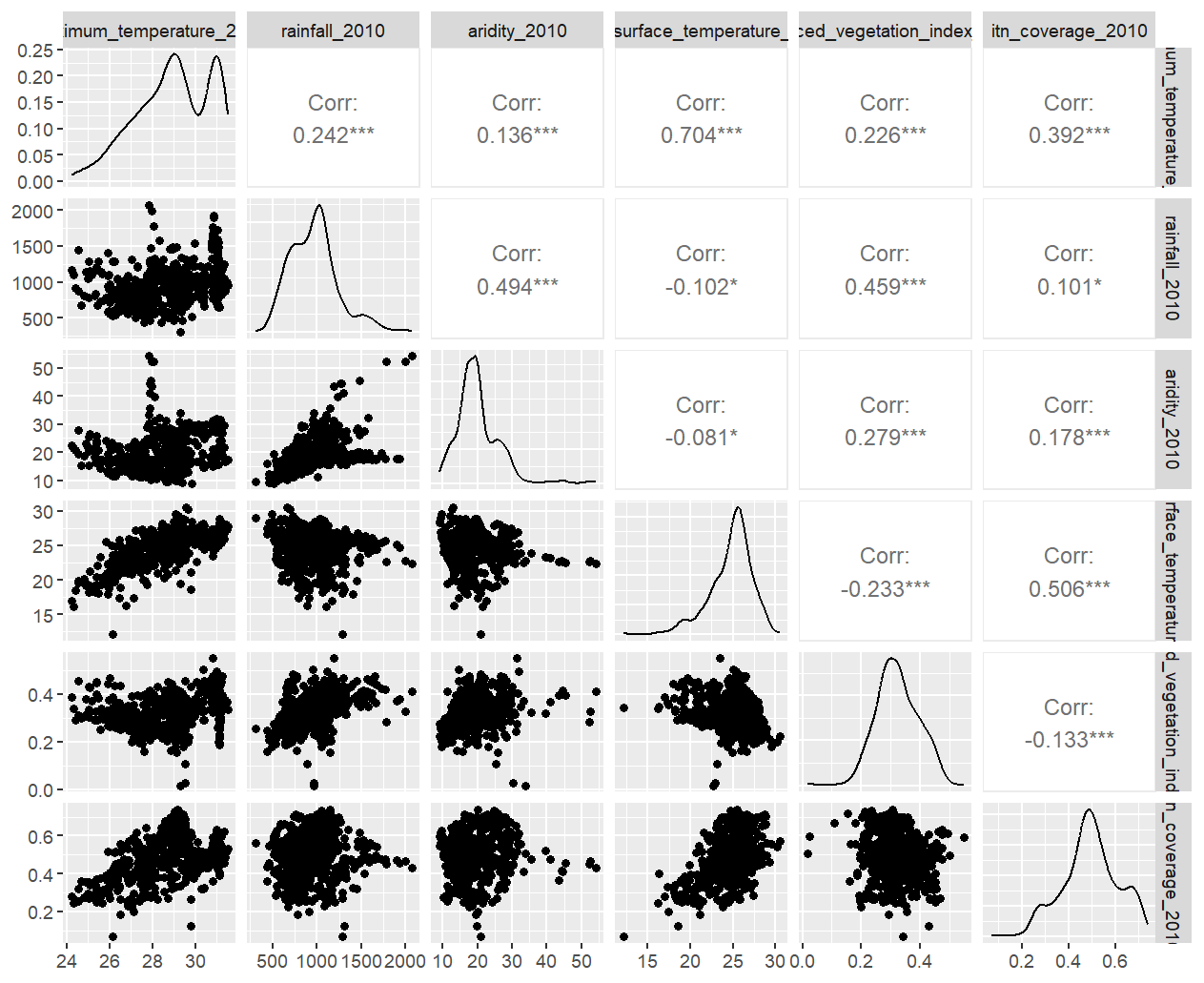
**

**S1B Fig.** Pairwise correlation of covariates for 2010.

**
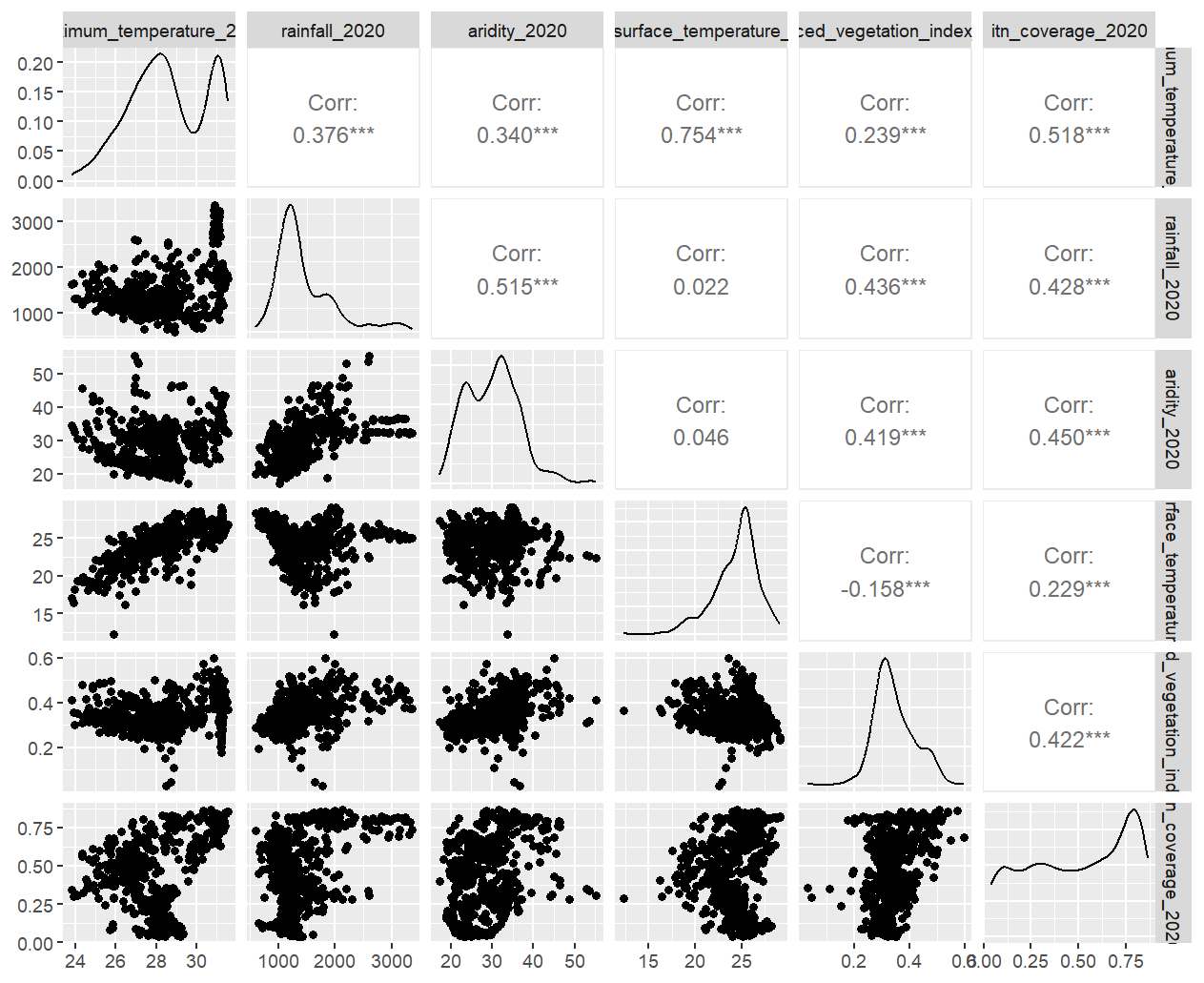
**

**S1C Fig.** Pairwise correlation of covariates for 2020.
