# Supplementary material for: Modelling the impact of climatic and environmental variables on malaria incidence in Tanzania: Implications for achieving the WHO’s 2030 Targets
Source: PLOS Glob Public Health. 2025 Aug 20;5(8):e0005075. doi: 10.1371/journal.pgph.0005075 (PMC12367119; doi:10.1371/journal.pgph.0005075)
Supplement: S2 File — Each circle represents a sampled cluster location for Rainfall (Row 1), and Maximum temperature (Row 2). S2B Fig. Spatial distribution of covariates. Each circle represents a sampled cluster location for Aridity (Row 1), and EVI (Row 2). S2C Fig: Spatial distribution of ITN coverage. Each circle represents a sampled cluster location for ITN coverage. (DOCX) [file pgph.0005075.s002.docx]

**
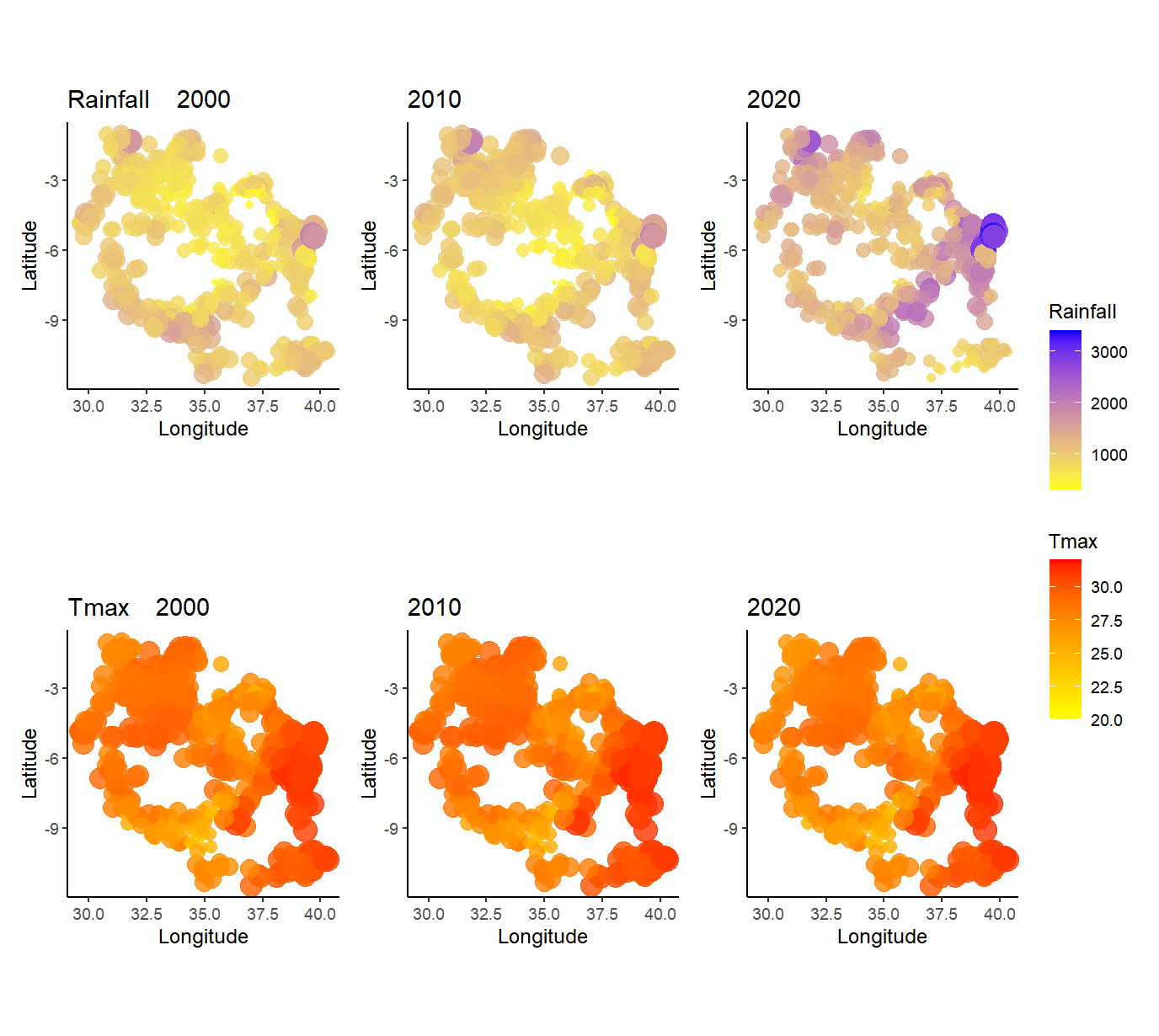
**

**S2A Fig.** Spatial distribution of covariates. Each circle represents a sampled cluster location for Rainfall (Row 1), and Maximum temperature (Row 2).

**
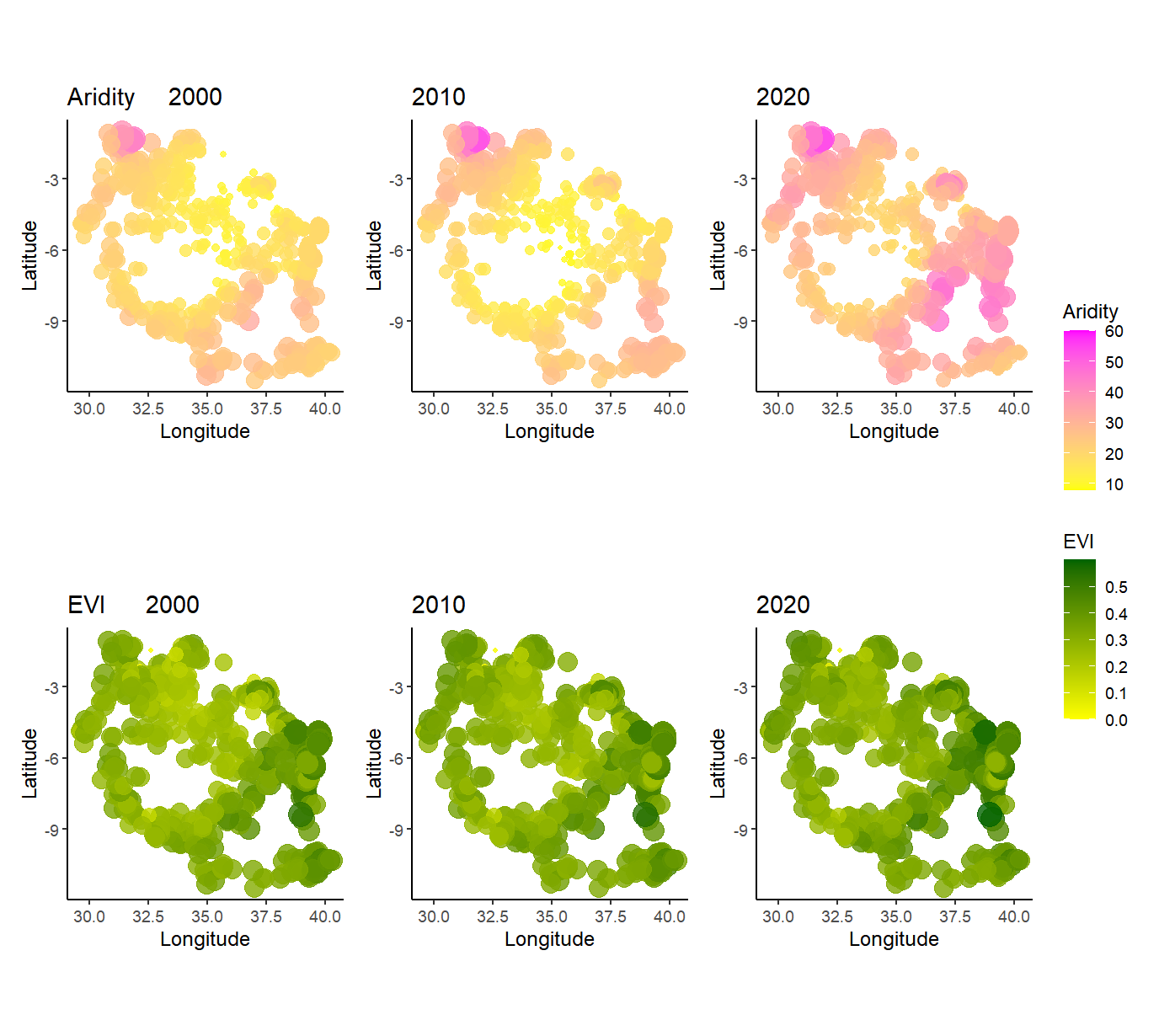
**

**S2B Fig.** Spatial distribution of covariates. Each circle represents a sampled cluster location for Aridity (Row 1), and EVI (Row 2).


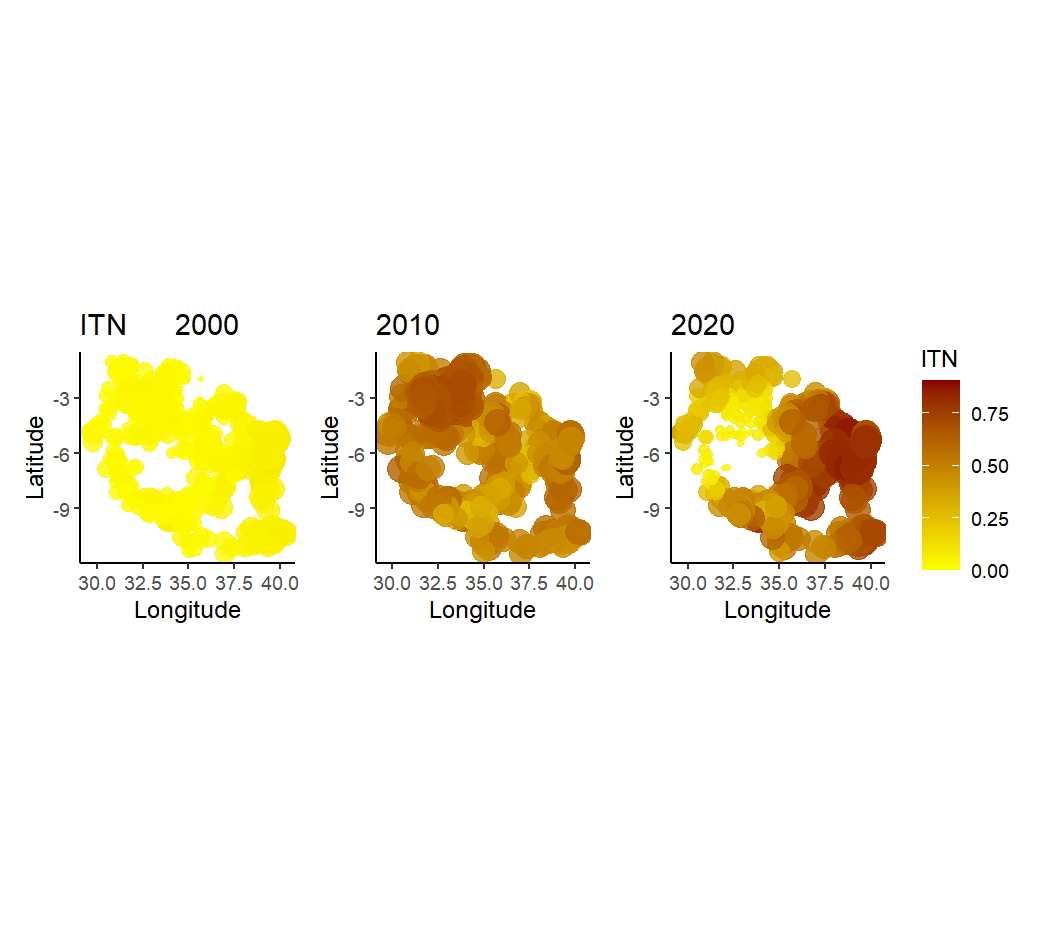


**S2C Fig.** Spatial distribution of ITN coverage. Each circle represents a sampled cluster location for ITN coverage.
